# Plant Diversity Surpasses Plant Functional Groups and Plant Productivity as Driver of Soil Biota in the Long Term

**DOI:** 10.1371/journal.pone.0016055

**Published:** 2011-01-07

**Authors:** Nico Eisenhauer, Alexandru Milcu, Alexander C. W. Sabais, Holger Bessler, Johanna Brenner, Christof Engels, Bernhard Klarner, Mark Maraun, Stephan Partsch, Christiane Roscher, Felix Schonert, Vicky M. Temperton, Karolin Thomisch, Alexandra Weigelt, Wolfgang W. Weisser, Stefan Scheu

**Affiliations:** 1 Department of Forest Resources, University of Minnesota, St. Paul, Minnesota, United States of America; 2 Division of Biology, NERC Centre for Population Biology, Imperial College London, Ascot, United Kingdom; 3 Institute of Zoology, Darmstadt University of Technology, Darmstadt, Germany; 4 Department of Plant Nutrition and Fertilization, Humboldt University of Berlin, Berlin, Germany; 5 J.F. Blumenbach Institute of Zoology and Anthropology, Georg-August-University Goettingen, Goettingen, Germany; 6 Department of Community Ecology, Helmholtz Centre for Environmental Research - UFZ, Halle, Germany; 7 IBG-2 Plant Sciences, Forschungszentrum Juelich GmbH, Juelich, Germany; 8 Department of Botany and Functional Biodiversity, Institute of Biology I, University of Leipzig, Leipzig, Germany; 9 Institute of Ecology, Friedrich-Schiller-University Jena, Jena, Germany; University of Zurich, Switzerland

## Abstract

**Background:**

One of the most significant consequences of contemporary global change is the rapid decline of biodiversity in many ecosystems. Knowledge of the consequences of biodiversity loss in terrestrial ecosystems is largely restricted to single ecosystem functions. Impacts of key plant functional groups on soil biota are considered to be more important than those of plant diversity; however, current knowledge mainly relies on short-term experiments.

**Methodology/Principal Findings:**

We studied changes in the impacts of plant diversity and presence of key functional groups on soil biota by investigating the performance of soil microorganisms and soil fauna two, four and six years after the establishment of model grasslands. The results indicate that temporal changes of plant community effects depend on the trophic affiliation of soil animals: plant diversity effects on decomposers only occurred after six years, changed little in herbivores, but occurred in predators after two years. The results suggest that plant diversity, in terms of species and functional group richness, is the most important plant community property affecting soil biota, exceeding the relevance of plant above- and belowground productivity and the presence of key plant functional groups, i.e. grasses and legumes, with the relevance of the latter decreasing in time.

**Conclusions/Significance:**

Plant diversity effects on biota are not only due to the presence of key plant functional groups or plant productivity highlighting the importance of diverse and high-quality plant derived resources, and supporting the validity of the singular hypothesis for soil biota. Our results demonstrate that in the long term plant diversity essentially drives the performance of soil biota questioning the paradigm that belowground communities are not affected by plant diversity and reinforcing the importance of biodiversity for ecosystem functioning.

## Introduction

Mankind faces multiple anthropogenic global environmental changes, which are now large enough to exceed the bounds of natural variability [1], [2]. One of the most significant consequences of contemporary global change is the rapid decline of biodiversity in many ecosystems [3]–[6]. This unprecedented biodiversity loss has generated concern over the consequences for ecosystem functioning and services, and prompted a multitude of studies [7], [8]. Most studies focused on the effects of diversity loss on single trophic levels or measures of ecosystem functioning, considerably less attention has been paid to the consequences of plant diversity loss for the performance of multiple trophic levels and ecosystem functions [8]–[11]. This is surprising since cascading effects of biodiversity loss may result in a vicious circle of diversity loss due to the interconnectance between ecosystem or foodweb components, and the incidence of soil feedback mechanisms [12]–[14].

Terrestrial grasslands are widespread model systems for investigating the biodiversity – ecosystem functioning relationship [7], [15]–[18]. Soil biota constitute a significant component of terrestrial ecosystems by governing essential ecosystem functions, such as decomposition and recycling of organic residues, and thereby primary productivity and plant community composition [19]–[21]. Moreover, soil biota comprise some of the most deleterious herbivores and pathogens often emerging as destructive pests [20], [22]. Although this knowledge provoked multiple studies on the relationship between aboveground (plant) and belowground diversity and functions, the significance of this relationship still is disputed [10], [23]–[25].

The question arises how plant diversity affects soil biota and why there is this lack of consistency and mechanistic understanding. For instance, there is an ongoing debate on the relevance of plant species richness *versus* that of plant species identity or presence of key plant functional groups for soil biota and functions [11], [23], [25], [26]. Since virtually all soil organisms are heterotrophs, and thus essentially rely on the quantity and quality of plant derived residues entering the soil subsystem [20], [27], [28], declining plant diversity, accompanied by deterioration of resource diversity, quantity and quality, is likely to impact the density, diversity and functioning of soil biota [10], [25]. Hooper et al. [10] suggested a step-by-step hypothesis how the diversity of primary producers results in higher belowground diversity assuming strong bottom-up control of biodiversity in soil communities. In essence, increased diversity of plant derived resources increases the diversity of decomposers and herbivores in soil, which in turn promotes the diversity of other components of the soil food web. Moreover, an additional causal relationship between above- and belowground diversity may arise from enhanced microhabitat diversity in complex plant communities [29]. Acknowledging the mixed evidence on the correlation between above- and belowground density and diversity, Hooper et al. [10] highlighted the need to acquire a mechanistic understanding of this relationship and ascribed this topic top research priority.

Most previous studies highlighted the significance of key plant functional groups for the performance of soil biota. Particularly legumes enhance the fertility of soils by N_2_ fixation and the input of high quality (nitrogen-rich) litter materials [24], [26], [30]–[32]. Positive impacts of plant diversity thus often have been ascribed to the increased probability of including legume species in more diverse plant communities [24], [26], [33], i.e. the selection or sampling effect of plant diversity [34], [35]. Moreover, increased performance of soil biota has been attributed to elevated primary productivity [11], [24].

Recent studies indicate that missing or inconsistent responses of soil biota to plant diversity may have been due to belowground legacy effects and the short-term character of most studies [25], [26], [36]. Indeed, most studies are based on snapshot measures and detailed investigations of soil biota in time are extremely scarce [26], [37]. Further, only few studies considered a wide range of taxonomic or functional groups of soil biota in a single experiment [11], [24], [26], [33], [36] complicating the comparability of results. In addition, the observed variability in the response of soil biota to plant diversity may have been due to the investigation of differential trophic positions relative to the manipulated level. Recent reports suggest that the effect of diversity loss decreases with the trophic distance from the manipulated level [8], [26].

In order to improve our mechanistic understanding of plant diversity effects on soil biota, we repeatedly sampled soil microorganisms and soil fauna in the framework of the Jena Experiment, a large grassland plant diversity experiment in Germany [18]. We intended to provide a comprehensive overview of plant community effects on multiple functional components of soil biota, ranging from short-term (two years after establishment) to long-term effects (after six years). Moreover, the Jena Experiment offers the unique possibility to independently explore the impacts of plant species richness (1–60 species), plant functional group richness (1–4 groups), and presence of key plant functional groups. The block design and the high replication of the Jena Experiment allow accounting for soil heterogeneity effects [18] and to delineate genuine diversity effects [10]. Determination of plant productivity measures above and below the ground further allows exploring the relevance of mere biomass effects. We hypothesized that (1) the role of plant diversity as driver of soil biota increases with time thereby exceeding that of the presence of key plant functional groups, and (2) changes in plant diversity effects depend on the functional affiliation of soil biota with decomposers responding slowest due to soil legacy effects.

## Materials and Methods

### Experimental setup

The study was conducted in the framework of the Jena Experiment, a large field experiment investigating the role of biodiversity for element cycling and trophic interactions in grassland communities [18]. The study site is located on the floodplain of the Saale river at the northern edge of the city of Jena (Thuringia, Germany). Mean annual air temperature 3 km south of the field site is 9.3°C and annual precipitation is 587 mm (Fig. S1; [38]). The site had been used as an arable field for the last 40 years and the soil is an Eutric Fluvisol. The experiment was established in May 2002 and the studied system represents Central European mesophilic grassland traditionally used as hay meadow (*Arrhenatherion* community). A pool of 60 native plant species was used to establish a gradient of plant species (1, 2, 4, 8, 16 and 60) and plant functional group richness (1, 2, 3 and 4) in a total of 82 plots of 20×20 m (Table S1; [18]). Using above- and belowground morphological traits, phenological traits and N_2_ fixation ability, plant species were aggregated into four plant functional groups: grasses (16 species), small herbs (12 species), tall herbs (20 species), and legumes (12 species) [18]. Experimental plots were mown twice a year (June and September), as is typical for hay meadows, and weeded twice a year (April and July) to maintain the target species composition. Plots were assembled into four blocks following a gradient in soil characteristics, each block containing an equal number of plots of plant species and plant functional group richness levels. Further information on the design and setup of the Jena Experiment is given in Roscher et al. [18].

### Soil biota

Soil samples for soil microbial measurements were taken from all plots in May 2004, 2006 and 2008. Briefly, at each sampling campaign, five soil samples were taken to a depth of 5 cm using a metal corer (diameter 5 cm), pooled and stored at 5°C. Before measurement, soil samples were homogenized, sieved (2 mm) to remove larger roots, animals and stones [39] and adjusted to a gravimetric soil water content of 25%. Microbial biomass C (C_mic_) was measured using an O_2_-microcompensation apparatus [40]. The microbial respiratory response was measured at hourly intervals for 24 h at 22°C. Substrate-induced respiration was calculated from the respiratory response to D-glucose [39]. Glucose was added according to preliminary studies to saturate the catabolic enzymes of microorganisms (4 mg g^−1^ dry weight solved in 400 µl deionized water). The mean of the lowest three readings within the first 10 h was taken as maximum initial respiratory response (MIRR; µl O_2_ h^−1^ g^−1^ soil dry weight) and microbial biomass (µg C g^−1^ soil dry weight) was calculated as 38 × MIRR [41].

Soil meso- and macrofauna were collected from soil cores taken to a depth of 10 cm in autumn 2004 (October), 2006 (November) and 2008 (October). Soil cores were taken using a steel corer (5 cm diameter for soil mesofauna, and 22 cm diameter for soil macrofauna). One soil core per plot for each meso- and macrofauna were taken (all 82 plots), and soil animals were extracted by heat [42], collected in diluted glycerol, and transferred into ethanol (70%) for storage. Soil animals were determined [43]–[49] and counted. A detailed list of soil animal taxa and their trophic assignment is given in Table S2. In the following, soil animal diversity is used as surrogate for the number of taxa per trophic group.

### Plant productivity

In order to explore if plant community effects are based on primary productivity, we considered data on plant shoot biomass (community biomass, cut 3 cm above soil surface level; see [50] for details) and plant root biomass (root diameter <2 mm, in 0–0.3 m soil depth; see [51] for details) in 2004, 2006 and 2008, respectively. These covariates were considered since changes in the performance of soil biota due to plant community effects have primarily been ascribed to plant productivity [11], [24], [52].

### Statistical analyses

Data generally were log-transformed to meet the requirements of ANOVAs (normal distribution and homoscedasticity of variances). Repeated measures analysis of variance as part of the general linear model (GLM, type I sum of squares) was used to analyze the effects of time (TI; if possible), block (BL; soil abiotic conditions), plant species richness (SR), plant functional group richness (FR), and presence of grasses (GR) and legumes (LE) on soil microbial biomass, the density of Collembola, Oribatida as well as on the density and diversity of macrofauna decomposers, herbivores and predators (in 2004, 2006 and 2008) in sequential analyses. We did not test the effects of the plant functional groups small and tall herbs due to negligible effects in pilot analyses (not shown). For the analysis of macrofauna density and diversity we used standardized values (minimum value  = 0, maximum value  = 1) in order present comparable results. We had to perform the analysis of soil macrofauna on the basis of trophic groups because low densities of single taxa did not allow for separate analyses (see Table S2 for the assignment of taxa to trophic groups). *F*-values given in text and tables refer to those where the respective factor was fitted first [53]. BL was always fitted first followed by plant community properties (SR, FR, GR, LE). Additionally, we tested if impacts of plant community characteristics rely on plant productivity by fitting plant shoot biomass and plant root biomass in 2004, 2006 and 2008 as covariates in separate analyses of covariance. Therefore, plant productivity measures were fitted after BL but before plant community properties. Moreover, we tested if plant diversity effects are solely based on the presence of key plant functional groups by considering the significance of plant diversity measures when fitted after plant functional groups. In order to investigate if plant species richness effects on decomposer animals (Collembola, Oribatida and macrofauna decomposers) are partly due to changes in microbial biomass as food source, we performed separate sequential GLMs by fitting microbial biomass before plant species richness.

We investigated the relevance of different plant community properties by comparing (1) the frequency of significant effects of plant community properties on soil biota (0 for non-significant, 1 for significant effects) using Cochran *Q* test, and (2) the *F*-values of effects of plant community properties on soil biota using Friedman ANOVA and Wilcoxon Matched Pairs Test. For this comparison we used the between-subject effects of the MANOVA results. These non-parametric tests are suitable for dependent variables and thus for the comparison of plant community properties. Additionally, we performed regressions between time (2004, 2006 and 2008) and the *F*-values of plant community properties (protected ANOVAs) in order to investigate if the relevance of different plant community properties changed over time. The analyses were performed using SAS V9.2 (SAS Institute Inc) and STATISTICA 7.1 (Statsoft). Means (± standard deviation) presented in Table S3 were calculated using non-transformed data.

## Results

Generally, impacts of soil heterogeneity (block) significantly affected several groups of soil biota and had to be considered in the statistical analyses, but are not presented and discussed in detail since this study focuses on plant community impacts on soil biota. A list of soil animal taxa of the field site of the Jena Experiment is given in Table S2 and mean values (± standard deviation) per taxon and year are given in Table S3. Data from single years of some animal taxa have been published previously (Collembola 2006, [54]; macrofauna 2008, Oribatida 2006, [55]).

### Soil microorganisms

**Figure 1:**
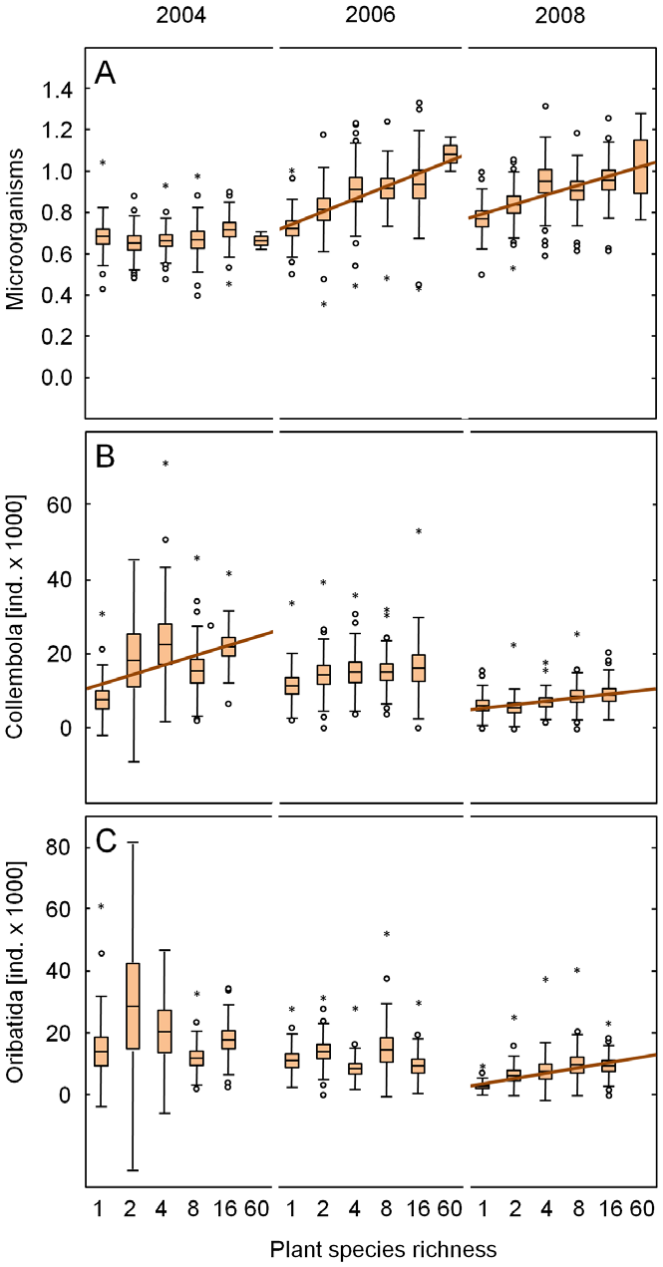
Plant species richness effects on soil microorganisms and mesofauna. Variations in (A) soil microbial biomass [mg C_mic_ g^−1^ soil dry weight], and the density of (B) Collembola, and (C) Oribatida [all individuals m^−2^] as affected by plant species richness in different years. Regression lines indicate significant effects (*P*<0.05); brown lines indicate decomposers in the broader sense (including microbivores); means (lines) with standard error (boxes), standard deviation (error bars), extremes (dots) and outliers (asterisks).

Annual data on the effects of plant community characteristics on microbial parameters [25] and effects of plant diversity on the mean microbial biomass of the years 2006, 2007 and 2009 [55] have been published elsewhere. Here data of 2004, 2006 and 2008 are included and analysed in the same way as data on soil fauna to allow a straightforward comparison of the response of these two major soil biota groups and in order to compare the relevance of plant community properties for soil biota. In general, soil microbial biomass increased significantly with plant species and functional group richness (Table 1), however, the impact of both plant diversity measures varied in time (Table S4). While soil microbial biomass was not affected significantly by plant diversity in 2004, it increased significantly with both plant diversity measures in 2006 and 2008 (Fig. 1A, Table 2). Moreover, microbial biomass was reduced in the presence of grasses (−8%), but increased in the presence of legumes (+10%) in 2004, whereas it was slightly increased in the presence of grasses (+2%) and legumes (+2%) in 2008 (Table 1, 2, S4).

**Table 1:** Between-subject factors effects.

		BL	SR	FR	GR	LE	*ER*
Microorganisms		16.12 ***	**16.17** ***	**16.81** ***	1.75	**7.60** **	*70*
Mesofauna							
	Collembola	0.73	**16.67** ***	**9.74** **	**4.55** *	**8.19** **	*66*
	Oribatida	0.65	**4.00** *	1.00	3.31	0.06	*66*
Macrofauna							
Density							
	Decomposers	8.55 ***	**7.55** **	**10.66** **	1.67	**4.61** *	*72*
	Herbivores	3.05 *	**14.65** ***	**15.05** ***	**6.67** *	**8.83** **	*71*
	Predators	1.30	**6.90** *	**10.79** **	**6.30** *	3.11	*72*
Diversity							
	Decomposers	3.94 *	**4.64** *	**4.87** *	0.26	3.21	*72*
	Herbivores	2.51	**22.13** ***	**22.64** ***	**11.85** **	**6.56** *	*71*
	Predators	1.41	**5.96** *	**6.89** *	**7.54** **	1.57	*72*

MANOVA (repeated-measures GLM) table of *F*-values of between-subject factors effects of Block (BL), plant species richness (SR), plant functional group richness (FR) and presence/absence of grasses (GR) and legumes (LE) on the biomass of microorganisms, the density of mesofauna as well as on the density and diversity of macrofauna decomposers, herbivores and predators.

Significant effects (*P*<0.05) of plant community properties are given in bold.

***P < 0.001,

**P<0.01,

*P<0.05. Degrees of freedom: BL = 3, SR, FR, GR, LE = 1 each. Error degrees of freedom (ER) are given in italics.

**Table 2:** Plant community effects in single years.

2004		BL	SR	FR	GR	LE	*ER*
Microorganisms		16.35 ***	0.35	1.31	**12.87** ***	**4.51** *	*70*
Mesofauna							
	Collembola	0.06	**18.28** ***	**10.96** **	**6.06** *	**8.96** **	*66*
	Oribatida	0.62	1.42	0.96	**4.52** *	0.62	*66*
Macrofauna							
Density							
	Decomposers	4.87 **	1.83	3.08	0.86	3.12	*72*
	Herbivores	3.14 *	**11.40** **	**11.16** **	0.01	0.89	*71*
	Predators	1.77	**4.77** *	**12.97** ***	0.03	0.28	*72*
Diversity							
	Decomposers	4.83 **	0.4	1.37	0.32	2.82	*72*
	Herbivores	1.35	**13.97** ***	**11.73** ***	0.01	1.1	*71*
	Predators	0.6	**11.35** **	**15.22** ***	0.11	0.06	*72*

2006							
Microorganisms		5.08 **	**12.02** ***	**8.07** **	0.12	0.6	*70*
Mesofauna							
	Collembola	4.04 *	0.12	0.28	0.09	2.12	*66*
	Oribatida	3.75 *	0.39	0.31	0.06	0.01	*66*
Macrofauna							
Density							
	Decomposers	1.23	0.84	2.28	0.57	**5.72** *	*72*
	Herbivores	0.98	0.98	**8.21** **	0.66	0.01	*71*
	Predators	1.63	1.46	3.09	1.21	0	*72*
Diversity							
	Decomposers	0.02	0.13	2.81	0.65	**5.55** *	*72*
	Herbivores	0.37	2.9	**9.06** **	1.46	0.46	*71*
	Predators	3.85 *	0.04	0.88	0.58	0.07	*72*

2008							
Microorganisms		13.94 ***	**14.03** ***	**18.90** ***	0.03	0.13	*70*
Mesofauna							
	Collembola	1.20	**4.56** *	1.98	0.65	0.43	*66*
	Oribatida	1.46	**6.56** *	1.64	2.68	0.00	*66*
Macrofauna							
Density							
	Decomposers	3.46 *	**6.79** *	**5.89** *	1.16	0.48	*72*
	Herbivores	3.38 *	**5.58** *	2.24	0.07	0.16	*71*
	Predators	8.07 ***	1.00	0.04	0.30	1.42	*72*
Diversity							
	Decomposers	2.26	**7.97** **	1.56	3.55	2.77	*72*
	Herbivores	5.48 **	**13.90** ***	**7.21** **	0.18	1.51	*71*
	Predators	6.25 ***	0.81	0.48	3.25	0.77	*72*

*F*-values of protected GLMs for the effects of Block (BL), plant species richness (SR), plant functional group richness (FR) and presence/absence of grasses (GR) and legumes (LE) on the biomass of microorganisms, the density of mesofauna as well as on the density and diversity of macrofauna decomposers, herbivores and predators in 2004, 2006 and 2008.

Significant effects (*P*<0.05) of plant community properties are given in bold.

***P<0.001,

**P<0.01,

*P<0.05. Degrees of freedom: BL = 3, SR, FR, GR, LE = 1 each. Error degrees of freedom (ER) are given in italics.

### Soil mesofauna

Here we focus on annual variations in Collembola density and on temporal changes of plant community effects, for seasonal variations in plant community effects on Collembola density and diversity at the Jena Experiment field site see [54]. Collembola density generally increased with increasing plant diversity (Table 1), although impacts of plant species richness varied in time (Table S4). The positive effect of plant species and functional group richness was most pronounced in 2004 (Fig. 1B), only true for plant species richness in 2008 and not present in 2006 (Table 2). However, it should be noted that in order to investigate plant community effects in time we had to exclude a number of plots which rendered the effect of plant diversity on Collembola density insignificant in 2006. Including spring and autumn samples in Sabais et al. [54] proved Collembola density to increase with plant diversity in 2006. The presence of plant functional groups affected Collembola densities significantly and largely consistently (Table 1, S4). However, the effects were most pronounced in 2004; Collembola densities were increased by +263% and +380% in the presence of grasses and legumes, respectively (Table 2).

Plant species richness increased the density of Oribatida (Table 1), although this was not consistent in time (Table S4) and only significant in 2008 (Fig. 1C, Table 2). Moreover, the density of Oribatida was increased significantly in the presence of grasses in 2004 (+111%; Table 2). The remaining plant community properties had no significant effect on the density of Oribatida (Table 1, 2, S4).

Fitting microbial biomass before plant species richness in additional sequential analyses rendered the effect of plant species richness on Collembola density insignificant (*F*_1,73_ = 6.10, *P* = 0.016 changed to *F*_1,72_ = 3.44, *P* = 0.068) and decreased the significance of the effects on Oribatida density in 2008 (*F*_1,73_ = 8.99, *P* = 0.004 changed to *F*_1,72_ = 5.06, *P* = 0.027).

### Soil macrofauna

**Figure 2:**
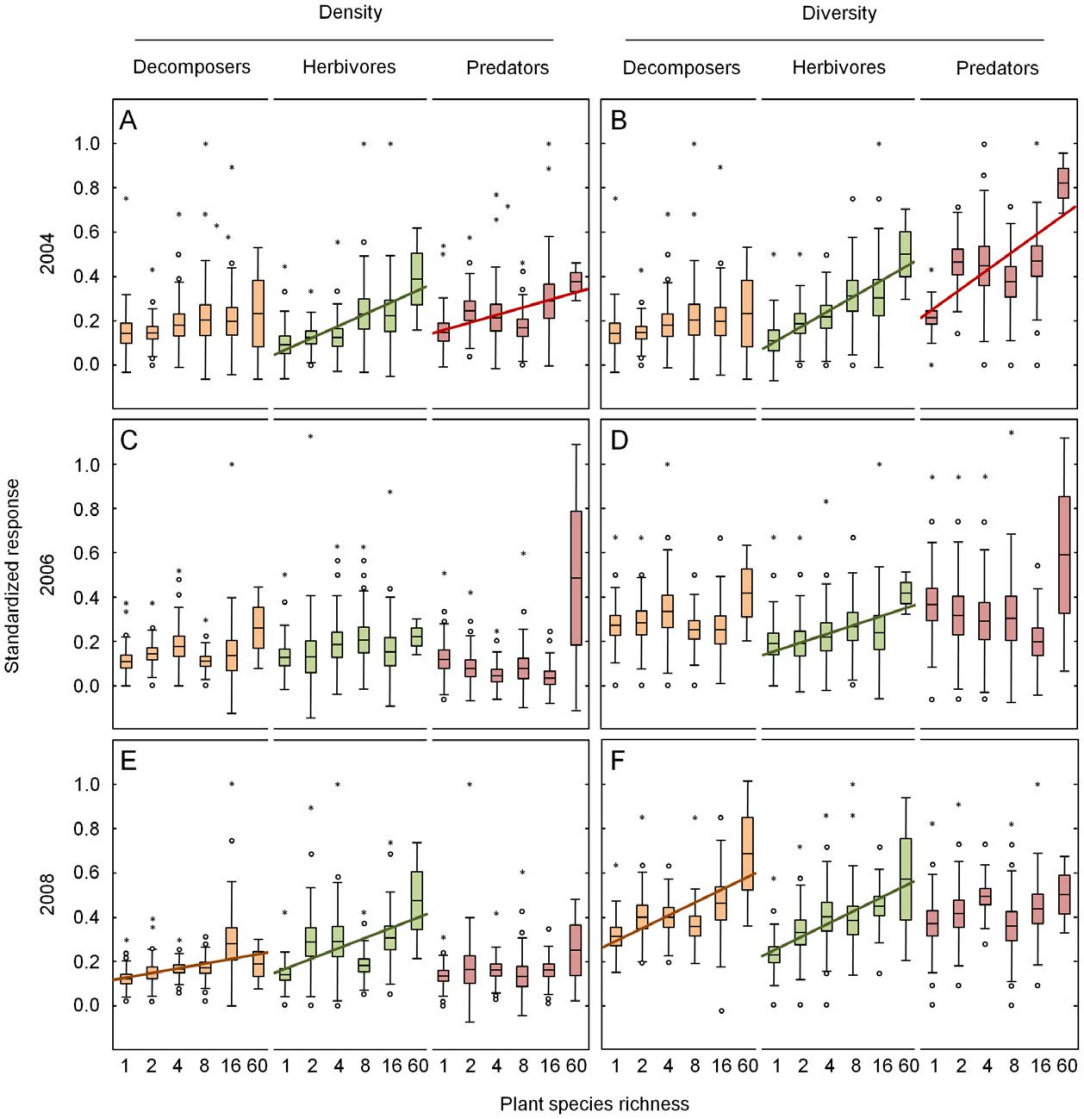
Plant species richness effects on soil macrofauna. Variations in the standardized (0 to 1) density (A, C, E) and diversity (B, D, F) of decomposers, herbivores and predators as affected by plant species richness in 2004 (A, B), 2006 (C, D) and 2008 (E, F). Regression lines indicate significant effects (*P*<0.05) and marginally significant effects (*P*<0.1; dashed line); brown lines indicate decomposers in the broader sense (including microbivores), green lines indicate herbivores, and red lines indicate predators; means (lines) with standard error (boxes), standard deviation (error bars), extremes (dots) and outliers (asterisks).

The density and diversity of decomposers was significantly affected by plant community properties (Table 1). While the impact of plant diversity measures on decomposer density and diversity was consistent in time that of legumes varied significantly (Table S4). In 2004 and 2006, plant community impacts on decomposer density and diversity were weak (Table 2). In 2004, decomposer density and diversity were increased by +93% and +44% in the presence of legumes, respectively. In 2006, there was the tendency of increased decomposer diversity with increasing plant functional group richness (Table 2). In 2008, decomposer density increased significantly with increasing plant species and functional group richness (Fig. 2E, Table 2). Moreover, decomposer diversity increased significantly with increasing plant species richness (Fig. 2F), but decreased in the presence of grasses (−9%). The remaining plant community properties did not significantly affect decomposer density and diversity (Table 1, 2).

Fitting microbial biomass before plant species richness in additional sequential analyses rendered the effect of plant species richness on decomposer density insignificant (*F*_1,71_ = 6.06, *P* = 0.016 changed to *F*_1,72_ = 2.77, *P* = 0.10) but did not change its effect on decomposer diversity in 2008 (*F*_1,71_ = 6.59, *P* = 0.012 changed to *F*_1,72_ = 6.03, *P* = 0.017).

The density and diversity of herbivores were generally affected by each of the plant community properties studied (Table 1). This was largely consistent in time, despite the tendency of varying plant species richness effect on herbivore density (Table S4). In 2004 herbivore density and diversity increased significantly with increasing plant species and functional group richness (Fig. 2A, B, Table 2). In 2006, this significant relationship was only true for plant functional group richness, while there was only the tendency of increased herbivore diversity with increasing plant species richness (Fig. 2C, D, Table 2). By contrast, in 2008 plant species richness was more important than plant functional group richness; herbivore density and diversity increased with increasing plant species richness, but only herbivore diversity increased significantly with increasing plant functional group richness (Fig. 2E, F, Table 2). The density and diversity of herbivores were increased in the presence of legumes by +32% and +41% in 2004, respectively, and in the presence of grasses by +13% and +36% in 2006, respectively (Table 2).

The density and diversity of predators was affected by plant species and functional group richness as well as by the presence of plant functional groups (Table 1). However, the effect of plant species richness on predator diversity, and the effect of plant functional group richness and legume presence on predator density and diversity varied significantly in time (Table S4). In 2004, predator density and diversity increased significantly with increasing plant species and functional group richness (Fig. 2A, B, Table 2). However, in 2006 there was only trend of increased predator density with increasing plant functional group richness (Table 2). In 2008, there was no significant plant diversity effect on predator density and diversity (Fig. 2E, F, Table 2). In 2004, predator density was increased by +28% in the presence of legumes, and predator diversity was increased by +43% and +41% in the presence of grasses and legumes, respectively (Table 2).

### Relevance of plant community properties

**Figure 3:**
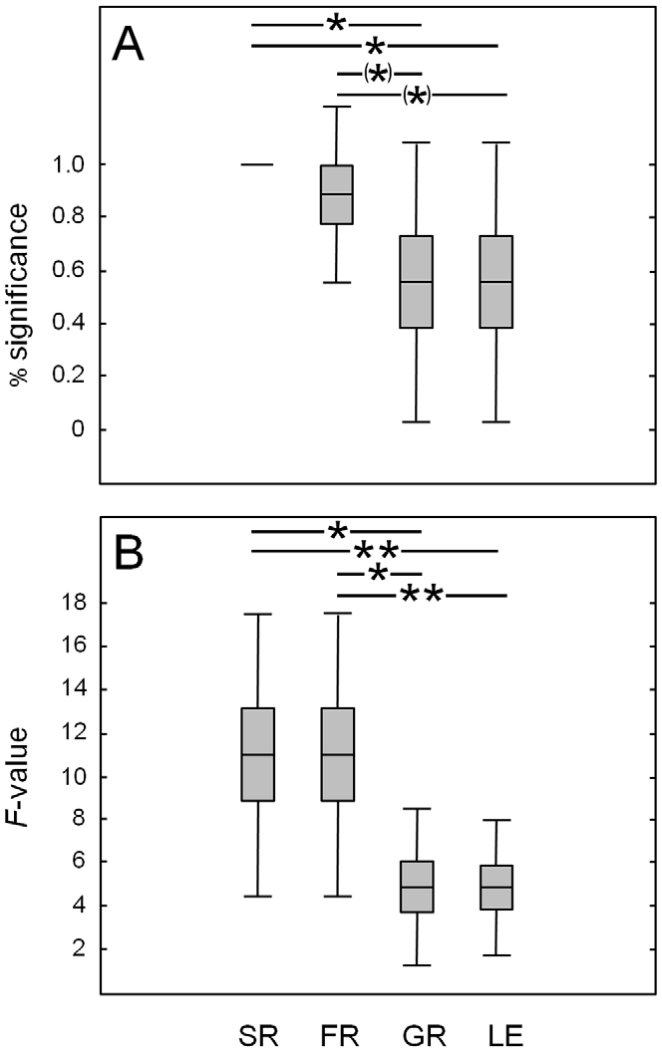
Relevance of plant community properties. (A) Percentage of soil biota variables significantly affected by plant species richness (SR), plant functional group richness (FR), presence of grasses (GR) and presence of legumes (LE). (B) Mean *F*-values of the effects of plant species richness, plant functional group richness, presence of grasses and presence of legumes on soil biota. Asterisks indicate significant (** *P*<0.01, * *P*<0.05) and marginally significant (^(^*^)^ *P*<0.1) effects. Means (lines) with standard error (boxes) and standard deviation (error bars).

Overall, the relevance of plant community properties for soil biota performance varied significantly (frequency (FQ): *Q* = 8.05, *P* = 0.045; *F*-values (FV): *Chi^2^* = 15.67, *P* = 0.0013). Plant species richness was more important for the performance of soil biota than presence of grasses (FQ: *Q* = 4.00, *P* = 0.046; FV: *Z* = 2.31, *P* = 0.021) and legumes (FQ: *Q* = 4.00, *P* = 0.046; FV: *Z* = 2.67, *P* = 0.008; Fig. 3). While the relevance of plant species richness and plant functional group richness did not differ significantly (FQ: *Q* = 1.00, *P* = 0.32; FV: *Z* = 0.89, *P* = 0.38), plant functional group richness was more important than the presence of grasses (FQ: *Q* = 3.00, *P* = 0.083; FV: *Z* = 2.31, *P* = 0.021) and legumes (FQ: *Q* = 3.00, *P* = 0.083; FV: *Z* = 2.67, *P* = 0.008; Fig. 3).

The relevance of plant species richness (*r* = 0.09, *P* = 0.66) and plant functional group richness (*r* = −0.29, *P* = 0.15) were largely consistent in time, whereas the relevance of the presence of grasses (*r* = −0.37, *P* = 0.058) and legumes (*r* = −0.53, *P* = 0.005) decreased significantly over time.

Fitting the presence of plant functional groups before plant species richness and plant functional richness hardly ever eliminated the significance of plant diversity measures (Table S5). Interestingly, plant species richness remained significant or marginally significant even when fitted after plant functional groups richness in affecting microbial biomass in 2006 (*F*_1,70_ = 4.67; *P* = 0.034), Collembola density in 2004 (*F*_1,66_ = 8.53; *P* = 0.005), Oribatida density in 2008 (*F*_1,66_ = 4.95; *P* = 0.030), macrofauna herbivore density in 2008 (*F*_1,71_ = 3.34; *P* = 0.072), macrofauna herbivore diversity in 2004 (*F*_1,71_ = 3.90; *P* = 0.051) and 2008 (*F*_1,71_ = 6.87; *P* = 0.011), and macrofauna decomposer diversity in 2008 (*F*_1,71_ = 7.03; *P* = 0.010).

### Relevance of plant productivity

Considering plant productivity measures in additional ANCOVAs showed that both plant shoot biomass and root biomass were not responsible for plant diversity effects in each of the trophic groups of macrofauna. Despite significant positive correlations between plant shoot biomass and the density and diversity of herbivores in 2004, the diversity of decomposers in 2008, that between plant root biomass and decomposer density in 2006, and a negative correlation between plant root biomass and the diversity of predators in 2006, fitting plant productivity measures as covariates hardly ever eliminated the significance of plant community properties (Table S5).

## Discussion

Conform to our first hypothesis, results of the present study indicate that the relative importance of major plant functional groups as drivers of soil biota decrease with time. In contrast, the importance of plant diversity remained rather constant, suggesting that in the long term plant diversity effects on soil biota are more important than the presence of key plant functional groups and plant productivity. Interestingly, the effect of plant species richness remained significant for several groups of soil biota even when fitted after plant functional group richness, suggesting that plant diversity effects on soil biota were not restricted to functional group richness.

### The relevance of plant community properties for soil biota

The superior role of plant diversity for soil biota as compared to plant productivity and the presence of key functional groups contrasts markedly to results of previous studies highlighting the predominant role of the presence of legumes [11], [24], [26], [33], [36], [37]. Recently, Eisenhauer et al. [25] stressed that plant diversity effects on soil biota have been underrated due to the short-term character of most experiments. They concluded that after a distinct belowground time-lag plant species richness is relevant for the performance and functions of soil microorganisms. Although legumes play an important role in temperate grassland by improving N availability [7], [30], [32], it has recently been shown that plant diversity effects on aboveground ecosystem functioning do not exclusively rely on the presence of legumes [56]–[58]. The results of the present study suggest a similar pattern for belowground responses. This may be due to the fact that soil biota do not solely rely on the availability of nitrogen but also on that of other nutrients such as phosphorous and micronutrients [30] pointing to the relevance of diverse plant derived inputs. Remarkably, plant diversity effects did not only rely on the functional diversity of plant assemblages confirming the findings by Reich et al. [58] on aboveground ecosystem functioning, and reinforcing the prominent role of plant species richness for soil processes.

Although based on a limited number of measurements, the results indicate that the decline in the role of plant functional groups for soil biota was due to decreasing impacts of grasses and legumes. In fact, the dominance of these two plant functional groups decreased with time: the mean percentage biomass of grasses in mixtures decreased from 46±6% in 2004 to 22±5% and 28±5% in 2006 and 2008, respectively, and the mean percentage biomass of legumes in mixtures decreased from 31±5% and 39±5% in 2004 and 2006, respectively, to 18±4% in 2008 [50]. Presumably, this was due to the fact that fast-establishing grass species benefited most from abundantly available soil nutrients after establishment of the experiment on a former highly fertilized agricultural field. Gradual depletion of soil phosphorous at the field site of the Jena Experiment (C. Roscher, unpubl. data) may be responsible for the decline of legumes over time.

### The mechanistic basis of plant diversity effects on soil biota

Virtually all groups of soil biota were positively affected by plant diversity in the present study, irrespective of trophic group affiliation. How can plant diversity promote and govern the performance of soil biota in such a pronounced way? Previous studies highlighted the relevance of mere biomass effects, suggesting that soil biota profit from elevated quantity of plant derived inputs [11], [52] which increase with plant diversity [7], [59], [60]. Results of recent and the present studies question this “quantity effect” and highlight the significance of quality of plant residue inputs [25], [55], [61], [62]. Interestingly, in the present study plant productivity measures were of minor importance both in the short and the long term. Plant residues enter the soil subsystem either *via* aboveground litter materials [20], [28] or *via* root litter and exudates [63], [64]. Again, root biomass effects poorly explained plant diversity effects on soil biota, and it was recently shown that both variables are not [11], [25] or even negatively correlated [37]; but see [65]. Thus, the key factor connecting above- and belowground diversity presumably is root-derived resources. Changes in root exudation can have pronounced effects on the structure of soil microbial communities [66] and hence on the soil food web. Remarkably, the amount of aboveground plant resources entering the belowground system likely is rather low due to the typical management of Central European mesophilic grasslands, in that plants are mown and the aboveground biomass removed twice a year. Milcu et al. [67] recently showed that plant diversity stabilizes belowground processes, presumably due to more consistent plant derived belowground inputs. The exploration of the composition of root-derived resources and their diversity represents a promising venue for achieving a mechanistic understanding of plant diversity effects on soil biota.

Microhabitat diversity may be another plant community characteristic promoting the performance of soil biota. Litter materials not only function as food but also as shelter, suggesting that both palatable and unpalatable litter materials may affect soil biota in a complementary way by enhancing food quality and physical protection [10]. Likewise, differences and diversity in root morphology may allow soil biota to populate the soil profile more completely than in simply structured environments. Thus, diversity in root morphology likely adds to belowground microhabitat richness.

### Temporal changes of plant diversity effects

Considering recent studies [25], [60], [68] we expected the significance of plant diversity effects to increase in time. This was not confirmed by the present experiment. However, while impacts on the density and diversity of decomposers occurred only six years after establishment of the model grasslands, those on herbivores were largely consistent in time, and those on predators were only significant after two years. Thus, hypothesis (2) is confirmed suggesting that changes in plant diversity effects depend on the functional affiliation of soil biota. Alterations in plant productivity above and below the ground were not responsible for the temporal changes in plant diversity effects (as indicated by fitting plant shoot and root biomass as covariates). As shown in a recent study by Eisenhauer et al. [25], microbial biomass and functions on the same field site strongly rely on the accumulation of dead plant materials and root exudates before plant community effects became manifest after a time lag of four years. The relevance of soil legacy effects on soil microorganisms is well established [12], [69], [70] and also applies for plant diversity effects on microorganisms changing from disturbed (zymogeneous) to more established (autochthonous) communities in grassland experiments [25], [69]. As decomposer animals also rely on plant derived resources entering the belowground subsystem [20], [27], the significant plant diversity effects on decomposer density and diversity in 2008 in the present study may have been driven by the accumulation of diverse plant residues and microbial communities. Unfortunately, the present study comprises too few measurements to adequately test if plant diversity effects on decomposers increased over time; however, the fact that microorganisms, Oribatida and macrofauna decomposer density and diversity only positively responded to plant diversity after a time-lag of four to six years point in this direction. Moreover, as many decomposer animals at least in part feed on soil microorganisms, we suggest a microorganism-mediated propagation of plant diversity effects to soil decomposers. Supporting this assumption, fitting microbial biomass before plant species richness in 2008 rendered the effects of plant species richness on the density of Collembola and macrofauna decomposers insignificant and reduced the significance of plant diversity effects on Oribatida. Additionally, the build-up of a root system [68] may have increased microhabitat diversity over time. The delayed response of decomposers to changes in plant diversity deserves further attention since it is likely to result in changes in soil feedbacks due to decomposer-mediated effects on nutrient cycling.

Belowground herbivores were strongly and consistently affected by plant diversity. The slight decrease of plant diversity effects in 2006 presumably was due to unusual dry weather conditions in June and July of this year (Fig. S1), generally resulting in low densities of soil animals and weak plant community effects. As herbivores directly consume fresh plant materials, impacts on plant diversity occurred almost without delay in 2004, i.e. two years after establishment of the experiment.

Generally, the majority of studies focussing on plant diversity effects on herbivores either investigated aboveground taxa [71]–[73], herbivory [56], [74], [75] or plant feeding Nematoda [26], [33], [36], [76]. Two non-mutually exclusive hypotheses exist explaining how herbivore densities are affected by plant diversity [77]. The “resource concentration hypothesis” predicts that densities of specialized herbivores decrease with plant diversity due to the dilution of host plants within a community of non-target plants (bottom-up effect). The “enemies hypothesis” assumes that natural enemies of herbivores are likely to be more abundant in diverse plant communities providing higher prey and refuge diversity as well as additional resources including pollen and nectar. Consequently, herbivore suppression by natural enemies should be more pronounced in diverse plant assemblages (top-down effect). Both hypotheses, therefore, predict lower herbivore density in diverse plant communities, albeit due to very different mechanisms. Experimental evidence for this negative relationship between plant diversity and herbivore performance, however, is scarce [72], [73].

Results of the present study contradict both of these hypotheses identifying belowground herbivores as the trophic group being most positively and consistently affected by plant diversity. Interestingly and similar to decomposers, plant biomass did not explain the increase in herbivore density and diversity in the short and the long term. We therefore conclude that the richness of microhabitats [26] and/or the availability and diversity as well as temporal stability of high-quality food resources [55], [67], [76] represent the most important plant community characteristics affecting belowground herbivore performance. This assumption is supported by the fact that the impact of plant diversity on the diversity of macro-invertebrate herbivores was more significant than that on herbivore density; pure biomass effects should have resulted in elevated herbivore density, while habitat or resource richness is likely to predominantly affect herbivore diversity. Potentially, the lack of conformity of the response of herbivores to the “resource concentration hypothesis” and the “enemies hypothesis” may also have been due to shifts in the herbivore community structure from specialists to generalists. Unfortunately, low numbers of macrofauna taxa did not allow more detailed analyses.

Although previous studies on soil nematodes, which comprise some of the most relevant soil herbivores, highlighted the relevance of plant identity effects rather than that of plant diversity [26], [76], a recent long-term study showed that effects of particular species vary in time [26]. Viketoft et al. [26] ascribed this increase in nematode diversity to sampling effects, particularly to the presence of the legume species *Trifolium pratense*. By contrast, our results argue for complementarity effects of plant diversity on herbivores since fitting the presence of plant functional groups before plant diversity measures reduced their significance only marginally.

In contrast to decomposers and herbivores, and our expectations, effects of plant diversity on the density and diversity of macro-invertebrate predators only occurred two years after establishment of the experiment. The initial positive relationship between plant diversity and predator performance may have been due to elevated prey (herbivore) density and diversity, with the mobility of most predator species allowing to respond fast to prey availability. However, the lack of plant diversity effects on predators in 2008 is difficult to interpret. Two factors, potentially acting in concert, may have resulted in this pattern. First, as argued by Gastine et al. [33] complex interactions between organisms obscure plant diversity effects on higher trophic levels. Prey availability was increased in more diverse plant communities in 2008, as both decomposer and herbivore densities increased with plant diversity. Potentially, elevated intraguild predation counteracted increased prey availability. Second, higher trophic levels not considered in the present study, such as higher-order (top) predators and parasitoids, may have reached high densities in diverse plant communities, thereby suppressing lower-order predators [78]. More long-term studies are needed to shed light on the mechanisms responsible for plant diversity effects on soil macro-invertebrate predators.

### Caveats

The efficiency of our sampling method for soil meso- and macrofauna depends on the mobility and ecology of soil animal taxa. Particularly immobile groups, such as Diptera larvae, were underrepresented and were thus not included in the analyses. Further, by taking only one soil core per plot and year for soil meso- and macrofauna we disregarded the role of soil heterogeneity and climatic conditions. For instance, low precipitation in June and July 2006 and resulting low densities of soil animals may have masked stronger plant community effects. Taking more samples per plot to more adequately represent the plant community composition of the plots was not possible due to the time necessary for taking and analyzing further samples. However, as the limited sampling design decreases the power of our statistical analyses due to elevated error variances we assume the results to be robust. Also, the large number of statistical tests could have inflated the chance of getting significant results, i.e. an increase of type I error. However, recently there is increasing criticism on the use of, for instance, the “Bonferroni correction” ranging from total refusal [79] to the suggestion of alternative, less conservative methods [80]. Moreover and as explained above, we believe that our approach represents a rather conservative measure of the consequences of plant diversity loss.

### Conclusions

In the present study plant diversity was the most important plant community trait affecting soil microorganisms and soil fauna. This impact did not rely on the presence of key functional groups or plant productivity, highlighting the relevance of diverse and high-quality plant derived resource inputs for the whole soil feed web. Moreover, plant diversity effects were not only restricted to functional richness, underlining the importance of plant species richness. We detected significant temporal changes of plant community effects with the relevance of key functional groups decreasing in time. Decomposers responded to plant diversity after a time-lag of four to six years with heretofore unknown long-term feedbacks to plants. In contrast to common view, the results suggest that plant diversity essentially drives the performance of soil biota and temporal changes in plant community effects need to be considered in order to adequately assess the relevance of plant community properties for ecosystem functioning.

## Supporting Information

Figure S1 **Temperature and precipitation from 2002 – 2008.** Mean temperatures (red line), minimum and maximum temperatures (orange lines) and total precipitation (blue line) per month in 2002 (start of the experiment), 2003, 2004 (first main sampling), 2005, 2006 (second main sampling), 2007 and 2008 (third main sampling) measured at the climatological station at the field site of the Jena Experiment.
(DOCX)

Table S1 **Design of the Jena Experiment.**
(DOCX)

Table S2 **List of soil animal taxa.**
(DOCX)

Table S3 **Mean density or biomass of soil organisms in 2004, 2006 and 2008.**
(DOCX)

Table S4 **Within‐subject factors effects.**
(DOCX)

Table S5 **Relevance of plant productivity and presence of plant functional groups.**
(DOCX)
