# Supplementary material for: Plant Diversity Surpasses Plant Functional Groups and Plant Productivity as Driver of Soil Biota in the Long Term
Source: PLoS One. 2011 Jan 7;6(1):e16055. doi: 10.1371/journal.pone.0016055 (PMC3017561; doi:10.1371/journal.pone.0016055)
Supplement: Figure S1 — Temperature and precipitation from 2002 – 2008. Mean temperatures (red line), minimum and maximum temperatures (orange lines) and total precipitation (blue line) per month in 2002 (start of the experiment), 2003, 2004 (first main sampling), 2005, 2006 (second main sampling), 2007 and 2008 (third main sampling) measured at the climatological station at the field site of the Jena Experiment. (DOCX) [file pone.0016055.s001.docx]

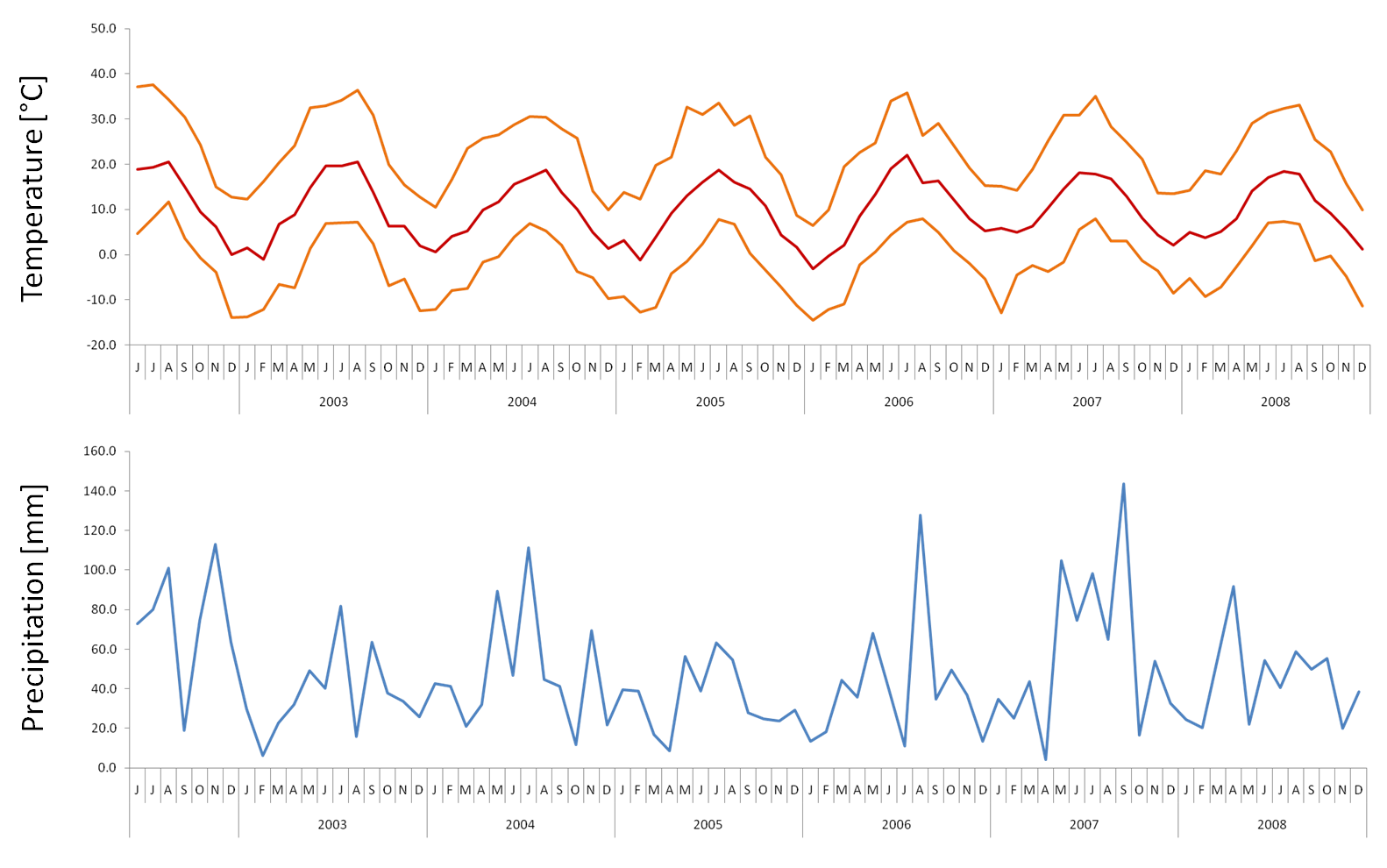


**Figure S1. Temperature and precipitation from 2002 – 2008.** Mean temperatures (red line), minimum and maximum temperatures (orange lines) and total precipitation (blue line) per month in 2002 (start of the experiment), 2003, 2004 (first main sampling), 2005, 2006 (second main sampling), 2007 and 2008 (third main sampling) measured at the climatological station at the field site of the Jena Experiment.
