# Supplementary material for: Plant Diversity Surpasses Plant Functional Groups and Plant Productivity as Driver of Soil Biota in the Long Term
Source: PLoS One. 2011 Jan 7;6(1):e16055. doi: 10.1371/journal.pone.0016055 (PMC3017561; doi:10.1371/journal.pone.0016055)
Supplement: Table S1 — Design of the Jena Experiment. (DOCX) [file pone.0016055.s002.docx]

**Table S1. Design of the Jena Experiment.** Combinations of plant species richness and plant functional group richness and the number of replicates per diversity level (given in italics). For more details on the experimental design see Roscher et al. [43].

|  |  | Plant species richness | | | | | | |  |
| --- | --- | --- | --- | --- | --- | --- | --- | --- | --- |
|  |  |  | 1 | 2 | 4 | 8 | 16 | 60 | Replicates |
| Plant functional group richness |  |  |  |  |  |  |  |  |  |
|  | 1 |  | *16* | *8* | *4* | *4* | *2* | *-* | *34* |
|  | 2 |  | *-* | *8* | *4* | *4* | *4* | *-* | *20* |
|  | 3 |  | *-* | *-* | *4* | *4* | *4* | *-* | *12* |
|  | 4 |  | *-* | *-* | *4* | *4* | *4* | *4* | *16* |
|  |  |  |  |  |  |  |  |  |  |
| Replicates | | *4* | *16* | *16* | *16* | *16* | *14* | *4* |  |
|  |  |  |  |  |  |  |  |  |  |
|  |  |  |  |  |  |  |  |  | 82 plots |
