# Supplementary material for: Plant Diversity Surpasses Plant Functional Groups and Plant Productivity as Driver of Soil Biota in the Long Term
Source: PLoS One. 2011 Jan 7;6(1):e16055. doi: 10.1371/journal.pone.0016055 (PMC3017561; doi:10.1371/journal.pone.0016055)
Supplement: Table S2 — List of soil animal taxa. (DOCX) [file pone.0016055.s003.docx]

**Table S2. List of soil animal taxa.** Meso- and macrofauna are grouped as either decomposers, herbivores, predators, omnivores or parasitoids according to existing literature (for details see the Material and Methods). We considered decomposers as litter, bacterial and fungal feeders (decomposers in the broader sense comprising primary and secondary decomposers). Omnivorous and parasitoid macrofauna were not considered in the present study due to inappropriate sampling methods and therefore low densities.

| Mesofauna | | |  | Macrofauna | | |
| --- | --- | --- | --- | --- | --- | --- |
| ▲ Decomposers (Collembola) | | |  | ▲ Decomposers | | |
|  | Entomobryidae | |  |  | Oligochaeta | |
|  |  | *Entomobrya lanuguinosa* |  |  |  | *Allolobophora chlorotica* |
|  |  | *Lepidocyrtus cyaneus* |  |  |  | *Aporrectodea caliginosa* |
|  |  | *Lepidocyrtus lanuginosus* |  |  |  | *Aporrectodea rosea* |
|  |  | *Lepidocyrtus paradoxus* |  |  |  | *Lumbricus terrestris* |
|  |  | *Lepidocyrtus curvicollis* |  |  |  | *Octolasion tyrtaeum* |
|  |  | *Heteromurus nitidus* |  |  | Isopoda | |
|  |  | *Pseudosinella alba* |  |  |  | *Armadillidium* sp. |
|  |  | *Pseudosinella immaculata* |  |  |  | Oniscidea |
|  |  | *Pseudosinella decipiens* |  |  | Diplopoda | |
|  |  | *Sinella coeca* |  |  |  | Blaniulidae |
|  | Isotomidae | |  |  |  | *Cylindroiulus* spp. |
|  |  | *Cryptopygus thermophilus* |  |  |  | Macrosternodesmidae |
|  |  | *Folsomides parvulus* |  |  |  | *Nemasoma* juvenile |
|  |  | *Isotoma* sp. |  |  |  | Polydesmidae juvenile |
|  |  | *Isotoma viridis* |  |  | Diplura | |
|  |  | *Isotomiella minor* |  |  |  | Campodeidae |
|  |  | *Isotomurus palustris* |  |  |  | Japygidae |
|  |  | *Isotomurus* sp. |  |  | Protura | |
|  |  | *Isotumurus fucicolus* |  |  |  | *Acerentomon* sp. |
|  |  | *Parisotoma notabilis* |  |  |  |  |
|  | Onychiuridae | |  | ► Herbivores | | |
|  |  | *Stenaphorura denisi* |  |  | Stylomatophora | |
|  |  | *Stenaphorura parisi* |  |  |  | *Arion* sp. |
|  |  | *Protaphorura quadriocellata* |  |  |  | *Deroceras* sp. |
|  |  | *Detriturus jubilarius* |  |  | Thysanoptera | |
|  | Hypogastruridae | |  |  |  | Thripidae |
|  |  | *Ceratophysella denticulata* |  |  | Coleoptera | |
|  |  | *Hypogastrura* sp. |  |  |  | Alticinae |
|  |  | *Willemia anophthalma* |  |  |  | *Batophila rubi* |
|  | Sminthuridae | |  |  |  | Byrrhidae |
|  |  | *Sminthurinus elegans* |  |  |  | Cholevidae |
|  |  | *Sminthurinus niger* |  |  |  | Curculionidae |
|  |  | *Stenacidia violacea* |  |  |  | Elateridae larvae |
|  |  |  |  |  |  | Scarabaeidae larvae |
| Other decomposer groups | | |  |  | Lepidoptera | |
|  |  | Enchytraeidae |  |  |  | Lepidoptera larvae |
|  |  | Oribatida |  |  | Heteroptera | |
|  |  | Symphyla |  |  |  | Tingidae |
|  |  |  |  |  | Sternorrhyncha | |
| ▼ Gamasid mites (predators) | | |  |  |  | Aphididae |
|  |  |  |  |  |  | Aphidina adult |
|  |  |  |  |  |  | Aphidina larae |
|  |  |  |  |  | Auchenorrhyncha | |
|  |  |  |  |  |  |  |
|  |  |  |  | ▼ Predators | | |
|  |  |  |  |  | Linyphiidae | |
|  |  |  |  |  |  | *Dicymbium nigrum* |
|  |  |  |  |  |  | *Diplocephalus cristatus* |
|  |  |  |  |  |  | *Eperigone trilobata* |
|  |  |  |  |  |  | *Erigone atra* |
|  |  |  |  |  |  | *Erigone dentipalpis* |
|  |  |  |  |  |  | *Micragus subaequalis* |
|  |  |  |  |  | Hahniidae | |
|  |  |  |  |  |  | *Hahnia nava* |
|  |  |  |  |  | Lycosidae | |
|  |  |  |  |  |  | *Pardosa* sp. |
|  |  |  |  |  |  | *Trochosa* sp. |
|  |  |  |  |  | Tetragnathidae | |
|  |  |  |  |  |  | *Pachygnatha degeeri* |
|  |  |  |  |  | Thomisidae | |
|  |  |  |  |  |  | *Ozyptila* sp. |
|  |  |  |  |  | Theridiidae | |
|  |  |  |  |  | Dictynidae | |
|  |  |  |  |  | Gnaphosidae | |
|  |  |  |  |  | Chilopoda | |
|  |  |  |  |  |  | Geophilidae |
|  |  |  |  |  |  | *Geophilus* sp. |
|  |  |  |  |  |  | Lithobiomorpha |
|  |  |  |  |  |  | *Lithobius microps* |
|  |  |  |  |  |  | *Lithobius* sp. |
|  |  |  |  |  |  | *Necrophloephagus longicornis* |
|  |  |  |  |  | Coleoptera | |
|  |  |  |  |  |  | Callicerini |
|  |  |  |  |  |  | Cantharidae larvae |
|  |  |  |  |  |  | Carabidae |
|  |  |  |  |  |  | Carabidae larvae |
|  |  |  |  |  |  | Staphylinidae |
|  |  |  |  |  |  | Staphylinidae larvae |
|  |  |  |  |  |  | *Tachyporus* sp. |
|  |  |  |  |  |  |  |
|  |  |  |  | ◄ Omnivores | | |
|  |  |  |  |  | Diptera | |
|  |  |  |  |  |  | Brachycera adult |
|  |  |  |  |  |  | Brachycera larvae |
|  |  |  |  |  |  | Cecidomyiidae larvae |
|  |  |  |  |  |  | Nematocera adult |
|  |  |  |  |  |  | Nematocera larvae |
|  |  |  |  |  | Hymenoptera | |
|  |  |  |  |  |  | Cynipidae |
|  |  |  |  |  |  | Dryinidae |
|  |  |  |  |  |  | Embolemidae |
|  |  |  |  |  |  | Formicinae |
|  |  |  |  |  |  | *Lasius flavus* |
|  |  |  |  |  |  | Myrmicinae |
|  |  |  |  |  | Heteroptera | |
|  |  |  |  |  |  | Cydnidae |
|  |  |  |  |  |  |  |
|  |  |  |  | ▼ Parasitoids | | |
|  |  |  |  |  | Hymenoptera | |
|  |  |  |  |  |  | Ceraphronoidea |
|  |  |  |  |  |  | Chalcidoidea |
|  |  |  |  |  |  | Myrmaridae |
|  |  |  |  |  |  | Platygastridae |
|  |  |  |  |  |  | Proctotrupoidea |
|  |  |  |  |  |  |  |
