# Supplementary material for: Plant Diversity Surpasses Plant Functional Groups and Plant Productivity as Driver of Soil Biota in the Long Term
Source: PLoS One. 2011 Jan 7;6(1):e16055. doi: 10.1371/journal.pone.0016055 (PMC3017561; doi:10.1371/journal.pone.0016055)
Supplement: Table S3 — Mean density or biomass of soil organisms in 2004, 2006 and 2008. (DOCX) [file pone.0016055.s004.docx]

**Table S3. Mean density or biomass of soil organisms in 2004, 2006 and 2008.** Means ± standard deviation of the microbial biomass C [µg g^-1^ soil dry weight] and the density of meso- and macrofauna [individuals m^-2^].

|  |  |  |  |  |
| --- | --- | --- | --- | --- |
|  |  | 2004 | 2006 | 2008 |
| Microorganisms | | 676 ± 130 | 872 ± 219 | 893 ± 194 |
|  |  |  |  |  |
| Mesofauna | |  |  |  |
|  | Collembola | 17108 ± 17745 | 14391 ± 9862 | 7500 ± 5813 |
|  | Oribatida | 18225 ± 28474 | 11286 ± 9951 | 7494 ± 7997 |
|  |  |  |  |  |
| Macrofauna | |  |  |  |
|  | Decomposers | 131 ± 153 | 104 ± 114 | 233 ± 201 |
|  | Herbivores | 38 ± 48 | 82 ± 135 | 127 ± 107 |
|  | Predators | 145 ± 138 | 96 ± 125 | 332 ± 321 |
|  |  |  |  |  |
