# Supplementary material for: Plant Diversity Surpasses Plant Functional Groups and Plant Productivity as Driver of Soil Biota in the Long Term
Source: PLoS One. 2011 Jan 7;6(1):e16055. doi: 10.1371/journal.pone.0016055 (PMC3017561; doi:10.1371/journal.pone.0016055)
Supplement: Table S4 — Within‐subject factors effects. (DOCX) [file pone.0016055.s005.docx]

**Table S4. Within-subject factors effects.** MANOVA table of *F*-values of within-subject factors effects of time (TI), and the interactions between TI and Block (BL), plant species richness (SR), plant functional group richness (FR) and presence/absence of grasses (GR) and legumes (LE) on the biomass of microorganisms, the density of mesofauna as well as on the density and diversity of macrofauna decomposers, herbivores and predators.

|  |  |  |  |  |  |  |  |  |
| --- | --- | --- | --- | --- | --- | --- | --- | --- |
|  |  | TI | TI x BL | TI x SR | TI x FR | TI x GR | TI x LE | *ER* |
| Microorganisms | | 57.04 *** | 4.03 *** | **5.19 **** | **3.39 *** | **6.98 **** | 0.47 | *140* |
|  |  |  |  |  |  |  |  |  |
| Mesofauna | |  |  |  |  |  |  |  |
|  | Collembola | 4.65 * | 1.62 | **5.01 **** | 2.73 | 1.67 | 1.94 | *132* |
|  | Oribatida | 12.32 *** | 2.77 * | **4.21 *** | 1.26 | 1.50 | 0.08 | *132* |
|  |  |  |  |  |  |  |  |  |
| Macrofauna | |  |  |  |  |  |  |  |
| Density | |  |  |  |  |  |  |  |
|  | Decomposers | 1.06 | 1.00 | 0.45 | 0.13 | 0.35 | **3.78 *** | *144* |
|  | Herbivores | 20.88 *** | 2.94 ** | 2.80 | 1.35 | 0.19 | 1.27 | *142* |
|  | Predators | 8.71 *** | 4.36 *** | 0.90 | **5.08 **** | 1.11 | **4.12 *** | *144* |
| Diversity | |  |  |  |  |  |  |  |
|  | Decomposers | 24.49 *** | 1.33 | 2.36 | 0.07 | 1.60 | **3.05 *** | *144* |
|  | Herbivores | 11.19 *** | 1.91 | 1.28 | 0.30 | 0.43 | 1.63 | *142* |
|  | Predators | 11.63 *** | 4.00 ** | **4.56 *** | **6.90 **** | 1.19 | **4.02 *** | *144* |
|  |  |  |  |  |  |  |  |  |

Significant effects (*P* < 0.05) of plant community properties are given in bold. *** *P* < 0.001, ** *P* < 0.01, * *P* < 0.05, ^(^*^)^ *P* < 0.1. Degrees of freedom: BL = 3, SR, FR, GR, LE = 1 each. Error degrees of freedom (ER) are given in italics.
