# Supplementary material for: Plant Diversity Surpasses Plant Functional Groups and Plant Productivity as Driver of Soil Biota in the Long Term
Source: PLoS One. 2011 Jan 7;6(1):e16055. doi: 10.1371/journal.pone.0016055 (PMC3017561; doi:10.1371/journal.pone.0016055)
Supplement: Table S5 — Relevance of plant productivity and presence of plant functional groups. (DOCX) [file pone.0016055.s006.docx]

**Table S5. Relevance of plant productivity and presence of plant functional groups.** *F*-values of ANCOVAs for the effects of Block (BL), plant shoot biomass (AB; covariate), plant fineroot biomass (FB), plant species richness (SR), plant functional group richness (FR) and presence of grasses (GR) and legumes (LE) on the density and diversity of macrofauna decomposers, herbivores and predators in 2004, 2006 and 2008. *F-*values of GLMs in which presence of plant functional groups was fitted before plant diversity measures in 2004, 2006 and 2008.

|  |  |  |  |  |  |  |  |  |  |  |  |  |  |  |  |  |
| --- | --- | --- | --- | --- | --- | --- | --- | --- | --- | --- | --- | --- | --- | --- | --- | --- |
|  |  | BL | *AB* | *BB* | SR | FR | GR | LE | *ER* |  | SR after GR | FR after GR |  | SR after LE | FR after LE | *ER* |
| **2004** |  |  |  |  |  |  |  |  |  |  |  |  |  |  |  |  |
| Density | |  |  |  |  |  |  |  |  |  |  |  |  |  |  |  |
|  | Decomposers | 4.60 * | *1.57* | *2.39* | 1.01 | 1.92 | 0.62 | 3.72 | *71* |  | 1.64 | 3.30 |  | 0.15 | 0.01 | *73* |
|  | Herbivores | 3.22 * | ***6.92 **** | *0.04* | **7.00 **** | **6.18 *** | 3.73 | 2.35 | *71* |  | **7.70 **** | **6.14 *** |  | **6.02 *** | 3.31 | *73* |
|  | Predators | 1.39 | *2.96* | *0.61* | 2.13 | **8.17 **** | **4.04 *** | **4.19 *** | *71* |  | 1.63 | **7.25 **** |  | 0.82 | 3.54 | *73* |
| Diversity | |  |  |  |  |  |  |  |  |  |  |  |  |  |  |  |
|  | Decomposers | 4.50 * | *2.49* | *0.06* | 0.02 | 0.51 | 0.06 | 2.18 | *71* |  | 0.28 | 1.44 |  | 0.04 | 0.04 | *73* |
|  | Herbivores | 1.79 | ***8.41 ***** | *0.16* | **8.92 **** | **5.71 *** | **4.19 *** | 1.59 | *71* |  | **7.63 **** | **4.10 *** |  | **7.10 **** | 3.12 | *73* |
|  | Predators | 0.47 | *2.27* | *0.77* | **9.10 **** | **11.65 ***** | **9.40 **** | 2.90 | *71* |  | **4.23 *** | **6.25 *** |  | **5.15 *** | **7.52 **** | *73* |
| **2006** |  |  |  |  |  |  |  |  |  |  |  |  |  |  |  |  |
| Density | |  |  |  |  |  |  |  |  |  |  |  |  |  |  |  |
|  | Decomposers | 1.31 | *2.54* | ***5.53 **** | 1.69 | 2.67 | 2.53 | 1.70 | *71* |  | 0.17 | 0.36 |  | 1.97 | **5.92 *** | *73* |
|  | Herbivores | 0.95 | *0.01* | *1.45* | 0.81 | **7.13 **** | **8.69 **** | 0.83 | *70* |  | 0.08 | 2.29 |  | 0.41 | **4.19 *** | *72* |
|  | Predators | 1.47 | *3.78* | *3.52* | 1.20 | 2.05 | **6.58 *** | 0.66 | *71* |  | 0.49 | 0.33 |  | 1.29 | 1.62 | *73* |
| Diversity | |  |  |  |  |  |  |  |  |  |  |  |  |  |  |  |
|  | Decomposers | 0.05 | *2.53* | *0.12* | 0.62 | 3.08 | 2.94 | 2.32 | *71* |  | 0.05 | 0.37 |  | 0.55 | **5.52 *** | *73* |
|  | Herbivores | 0.50 | *0.07* | *1.59* | 2.64 | **7.81 **** | **11.03 **** | 0.00 | *70* |  | 0.91 | 1.89 |  | 2.85 | **6.80 *** | *72* |
|  | Predators | 3.28 * | *0.00* | ***5.95 **** | 0.20 | 0.15 | **4.65 *** | 0.39 | *71* |  | 0.34 | 0.00 |  | 0.07 | 0.29 | *73* |
| **2008** |  |  |  |  |  |  |  |  |  |  |  |  |  |  |  |  |
| Density | |  |  |  |  |  |  |  |  |  |  |  |  |  |  |  |
|  | Decomposers | 3.11 * | *0.27* | *0.16* | **5.91 *** | **5.18 *** | 0.35 | 2.00 | *69* |  | **6.31 *** | **6.44 *** |  | 3.68 | 2.20 | *71* |
|  | Herbivores | 3.11 * | *2.06* | *0.09* | 3.69 | 1.08 | 0.18 | 0.09 | *69* |  | 3.83 | 1.08 |  | 3.28 | 1.50 | *71* |
|  | Predators | 7.56 *** | *0.20* | *1.33* | 1.08 | 0.04 | 0.02 | 1.53 | *69* |  | 0.54 | 0.42 |  | 1.77 | 0.17 | *71* |
| Diversity | |  |  |  |  |  |  |  |  |  |  |  |  |  |  |  |
|  | Decomposers | 2.11 | ***7.22 ***** | *0.02* | **4.57 *** | **5.57 *** | 0.96 | 3.35 | *69* |  | **10.85 **** | **4.58 *** |  | **4.58 *** | 0.00 | *71* |
|  | Herbivores | 5.07 ** | *0.03* | *0.14* | **13.86 ***** | **6.87 *** | 3.63 | 0.04 | *69* |  | **8.49 **** | 2.12 |  | **13.37 ***** | **7.65 **** | *71* |
|  | Predators | 4.35 ** | *0.01* | ***4.47 **** | 0.84 | 0.52 | 0.18 | 1.55 | *69* |  | 0.01 | 2.91 |  | 0.66 | 0.16 | *71* |
|  |  |  |  |  |  |  |  |  |  |  |  |  |  |  |  |  |

Significant effects (*P* ≤ 0.05) of plant community parameters are given in bold. *** *P* ≤ 0.001, ** *P* ≤ 0.01, * *P* ≤ 0.05, ^(^*^)^ *P* ≤ 0.1. Degrees of freedom: BL = 3, AB, BB, SR, FR, GR, LE = 1 each. Covariates (AB and BB) and error degrees of freedom (ER) are given in italics.
